# Bio-inspired benchmark generator for extracellular multi-unit recordings

**DOI:** 10.1038/srep43253

**Published:** 2017-02-24

**Authors:** Sirenia Lizbeth Mondragón-González, Eric Burguière

**Affiliations:** 1 Sorbonne Universités, UPMC Univ Paris 06, CNRS, INSERM, Institut du cerveau et de la moelle épinière (ICM), F-75013 Paris, France

## Abstract

The analysis of multi-unit extracellular recordings of brain activity has led to the development of numerous tools, ranging from signal processing algorithms to electronic devices and applications. Currently, the evaluation and optimisation of these tools are hampered by the lack of ground-truth databases of neural signals. These databases must be parameterisable, easy to generate and bio-inspired, i.e. containing features encountered in real electrophysiological recording sessions. Towards that end, this article introduces an original computational approach to create fully annotated and parameterised benchmark datasets, generated from the summation of three components: neural signals from compartmental models and recorded extracellular spikes, non-stationary slow oscillations, and a variety of different types of artefacts. We present three application examples. (1) We reproduced *in-vivo* extracellular hippocampal multi-unit recordings from either tetrode or polytrode designs. (2) We simulated recordings in two different experimental conditions: anaesthetised and awake subjects. (3) Last, we also conducted a series of simulations to study the impact of different level of artefacts on extracellular recordings and their influence in the frequency domain. Beyond the results presented here, such a benchmark dataset generator has many applications such as calibration, evaluation and development of both hardware and software architectures.

Electrical recording of extracellular action potentials is the “gold standard” technique widely used in electrophysiology^1^, where the signals are exploited to correlate neural activity with a behavioural output and/or the electrophysiological consequences of brain lesions or drug infusion, etc. The emergence of novel methods for neural analysis together with high-throughput data acquisition technologies^2^ provide new possibilities for the exploitation of brain activity at the single unit level, for example, giving instantaneous feedback for closed-loop interactions with brain circuits when abnormal neural signals are detected^3^. This approach has proven effective for several pathological conditions such as Epilepsy, Parkinson’s disease, or Essential Tremor^4^,^5^,^6^,^7^. From a more fundamental perspective, novel algorithms have been recently proposed to process these large amounts of neural data, such as semi-automatic and automatic clustering techniques, to distinguish different neural sources in multi-unit extracellular recordings^8^,^9^,^10^,^11^,^12^. In order to validate the performance and accuracy of these different algorithms or devices, reliable datasets, where the majority of the signal content is known, are essential. Ideally, this ground-truth reference should be a completely annotated and parameterised dataset, in which three levels of information should be modifiable and known in detail: the recording environment (e.g. density of active population of neurons or distance from neurons to recording sites), the population dynamics (e.g. firing rate, spike timing of each neuron and spike waveforms) and the noise content (e.g. background noise level contribution and number of artefacts).

There are several applications (Fig. 1) where using a parameterised dataset can be advantageous, ranging from algorithm design to development and evaluation of electronic devices. Moreover, parameterised datasets are needed to evaluate the efficiency of unsupervised classification algorithms. In recent years, several spike sorting algorithms have been proposed^8^,^9^,^10^,^11^,^12^, however, it is difficult to assess their sorting efficiency since the datasets used to evaluate their performance were heterogeneous. These studies either used real recording datasets where all the events that constitute the signal were not known, or simulated datasets that did not include all the features encountered in real recording, such as slow oscillations and/or disturbance by artefacts. Therefore, one solution could be to use a fully annotated and parameterised dataset as a ground-truth reference to objectively assess the performance of these different spike sorting algorithms (Fig. 1a). In the same manner, fully annotated datasets could also be used to challenge event detectors or noise reduction algorithms (Fig. 1b and c).

In addition, these benchmarks could be very useful for brain-computer interfaces and neural prosthetic devices (Fig. 1d and e). The common approach to assess the performance of such electronic devices is to use a large number of neural signal datasets that include a range of various features (e.g. different noise levels, a degree of meaningful information load, signal resolution etc.). For this purpose, parameterised datasets with independently modifiable features would allow the generation of a large variety of neural signal profiles in a controlled manner. This approach could also enable the simulation of experiments for calibration purposes instead of performing labour- and cost-intensive experiments with real subjects.

Several approaches, based either on biological or purely computational models, have been proposed^13^ to generate reliable (in terms of biological constraints), fully annotated, and flexible benchmarks. With *in-vitro* biological approaches^14^,^15^, investigators have conducted simultaneous recordings to capture intracellular signals emitted by some neurons located closely to extracellular electrodes. Although this approach relies on real experimental data, the limitation is that only a few neurons could be followed by the intracellular recordings, which represent a small part of the complex signal recorded at the contiguous extracellular recording sites.

Computational approaches use either compartmental or biophysically data-driven models^13^,^16^,^17^,^18^,^19^,^20^,^21^,^22^,^23^,^24^. The former are parameterisable but computationally too demanding when required to simulate a large number of neurons. The latter, in contrast, are computationally simpler and faster but not parameterisable given the use of signal templates.

A more recent solution is a hybrid approach, where compartmental and biophysically data-driven models are combined: while the compartmental models serve to generate the neural signal, spike template-based models, on the other hand, simulate the physiological background noise^25^. Models based on this approach are a good compromise between complexity and bio-realism. Their great potential relies on their ability to generate a simulated signal similar to that arising from a large population of single neurons, leading to a more realistic approach. These hybrid models could be improved by adding other features found in experimental recordings such as corrupting events that could affect signal quality.

In the present study, we propose a computational procedure to generate realistic neural signals based on a hybrid model approach, in which both real and simulated signal features are combined with a relatively low computational requirement. The generated datasets are fully parameterisable and include all the original features found in real recordings such as a variety of different types of artefact and background noise. The validation stage of our procedure explores the similarity between real recordings and our model-generated signals. We show that our model is easily modifiable and generates synthetic signals similar to those obtained in distinct experimental conditions. We also illustrate the flexibility of our simulator by modelling different types of recording configuration (tetrodes and microelectrode arrays), brain tissue (such as juxtaposed layers) and experimental conditions (awake or anaesthetised animals). To validate our approach, we focus on reproducing hippocampal recording datasets that have been extensively used in previous studies^14^,^26^. With our parameterisable bio-realistic procedure, we can also easily simulate different experimental conditions. As an example, we show the incidence of different levels of artefact in anaesthetised or awake animals.

## Results

### Creation of a three module simulator of extracellular multi-unit signals

Our work proposes a computational procedure to generate datasets that will provide neuroscientists with a ground-truth reference for algorithm and tool evaluation of single and multi-unit signal processing. In our approach, ground-truth from real and simulated signals is obtained by adding spike activity, that is, action potentials from nearby neurons and background noise from distant neurons (*x* (*n*)), slow oscillations (<300 Hz) from synaptic current inputs (*w* (*n*)) and artefacts (*a* (*n*)) that can be expressed as:


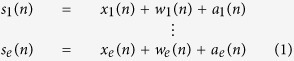


In equation (1) *s*_1_ (*n*) refers to bio-inspired simulation of electrode number 1, with *n* as discrete time variable and suffix *e* as the total number of simulated electrodes.

**Figure 2:**
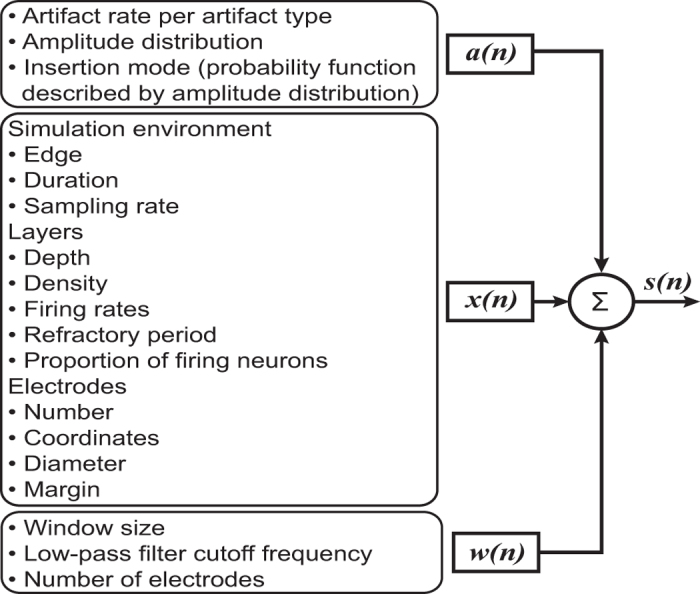
Configuration parameters of the benchmark dataset generator. For each computing module in the benchmark dataset generator, the user has rapid access to allow its modification through a unique file descriptor (.xml file) to easily create different datasets. The parameters are listed inside the text boxes next to each computing module.

Figure 2 summarises the general approach and highlights the modifiable parameters in each individual computing module. The flexibility of this approach is reflected in the creation of different benchmark datasets by simply adjusting the simulation parameters.

### Comparison of simulated and real extracellular hippocampal recordings

**Figure 3:**
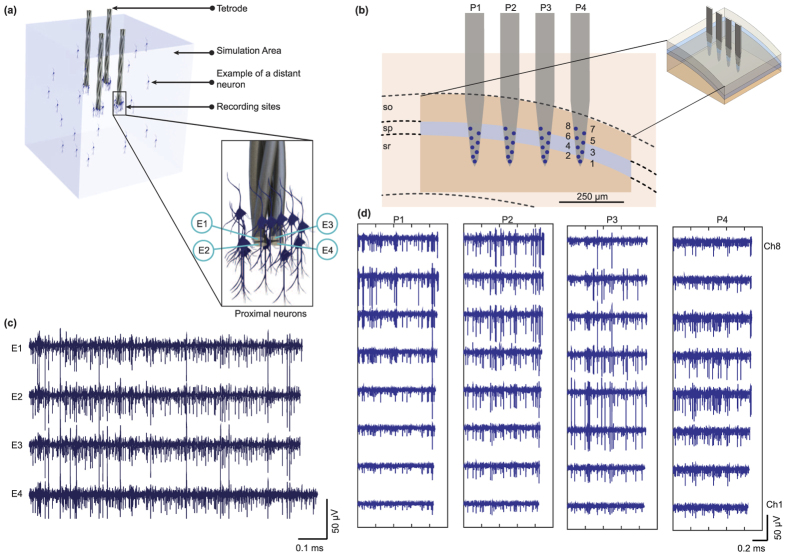
A data-driven model of the neural signal simulator. (**a**) The extracellular action potential simulator was modified from^25^ to integrate several virtual recording sites (i.e. 4 tetrodes) with different configurations of hippocampal neural populations. The model computes the extracellular action potential waveforms using detailed compartment models of pyramidal cells for nearby neurons and uses action potential templates from real recordings to simulate distant neurons. The result is the contribution of close and distant neurons using the line source approximation (LSA) method^58^ in a 3D virtual volume of tissue. (**b**) Modelling of a portion of virtual rat hippocampus with a 32-channel polytrode array (8 sites x 4 shanks, dimensions follow Buszaki64 probe from NeuroNexus) superimposed across (following a stereotaxic view) the stratum radiatum (sr), stratum pyramidale (sp) and stratum oriens (so) in the CA1 region of rat hippocampus. Data for dimensions and contours of rat hippocampal regions were measured from Swanson’s rat brain atlas to determine the dimensions of our volume, particularly between the atlas levels 30- = 35. A parasagittal view in the Figure shows atlas level 31^59^ (AP = −3.70 mm relative to Bregma, ML 2–3 mm, DV 2–3 mm). (**c**) Signal samples from one extracellular virtual tetrode. (**d**) Polytrode simulation datasets over one second are shown for each virtual polytrode. The activity across channels reflects the signal location of the virtual recording sites.

As a starting point, we created the contribution of the local spike activity to the signal. For this, we modified an existing simulation platform^25^ designed for a single multi-electrode (i.e. tetrode) to include multiple spatially distinct recording sites. The existing simulator implements a hybrid model that combines detailed compartment models of pyramidal cells and interneurons^18^,^27^,^28^,^29^,^30^,^31^ (available via the NEURON^32^ project) for the closest neurons to the recording sites, coupled with spike templates for the distant neurons, all in a 3D volume of “*virtual tissue”* (Fig. 3a).

The initial hybrid model^25^ that generated the spiking activity and background noise also gave the user, via a graphical interface, the option to modify various parameters to generate the datasets. These options allowed the user to select: a single electrode or a tetrode, a uniform (between a minimum and maximum firing rates) or exponential (generalised Pareto) distribution of firing rates, and a proportion of active cells inside a cubic volume. This hybrid model^25^ was improved in our approach by including any number of recording sites with specific coordinates in a volume of “virtual tissue”. We added the possibility to simulate multiple contiguous tissue volumes (e.g. cortical layers) with individual configurations and the possibility for the user to add customised firing rate distributions by using the Distribution Fitting App in Matlab^33^. These modifications gave more flexibility to the original model and enabled us to simulate different experimental scenarios. As an example, we simulated a recording session with a multi-electrode (polytrode) array in a virtual volume containing different neuronal populations in the hippocampus. We targeted the stratum oriens (SO), the stratum pyramidale (SP) and the stratum radiatum (SR) layers of the dorsal CA1 region of the rat hippocampus with a multi-array of 32 channels (8 channels × 4 shanks). The design of the spatial distribution of the recording sites were inspired by the Neuronexus “Buzsaki64” probe design (Fig. 3b). The virtual probes were positioned so that the recording sites were present across the three different layers. The characteristics of the neuronal population in each layer (in terms of overall firing rate, proportion of active neurons and neuron density) were determined by following the results reported in previous studies. Details of the configuration parameters for this experimental condition are summarised in Table 1 and in the Methods section. As shown in Fig. 3c the recording sites with the largest action potentials follow the spatial curve of the middle striatum pyramidal layer.

The next feature of our model designed to ensure that simulations were close to real experimental signals was to add the contribution of non-stationary slow oscillations. In the real world an experimenter starts to work with unfiltered raw data before applying further analysis. Those slow oscillations usually refer to the low-frequency part of an extracellular voltage signal recorded inside the brain. We extracted slow oscillations (<300 Hz) from real datasets containing extracellular multichannel recordings made from the CA1 hippocampal region of rats^14^,^26^,^34^,^35^ (and added them linearly to the neural simulations as the non-stationary low-frequency components^36^. Given that local field potential (LFP) < 300 Hz can be contaminated by action potentials, we were aware that the extraction of low frequency components require a preceding exploration^37^ on the original data to reveal the degree of spike contamination in LFP. In our case, we verified that the contribution of the spectral density of the mean spike waveform was negligible at low frequencies (≲300 Hz).

One element that is often omitted while developing realistic neural simulators is the inclusion of artefacts that contaminate real microelectrode recordings. In any extracellular data analysis, these undesired features should be considered, especially when it comes to unsupervised methods. Many algorithms of detection and artefact suppression have been previously reported, and they all require ground-truth data for evaluation and optimisation purposes. Hence, the inclusion of identified artefacts plays an important role in our neural database creation procedure.

**Figure 4:**
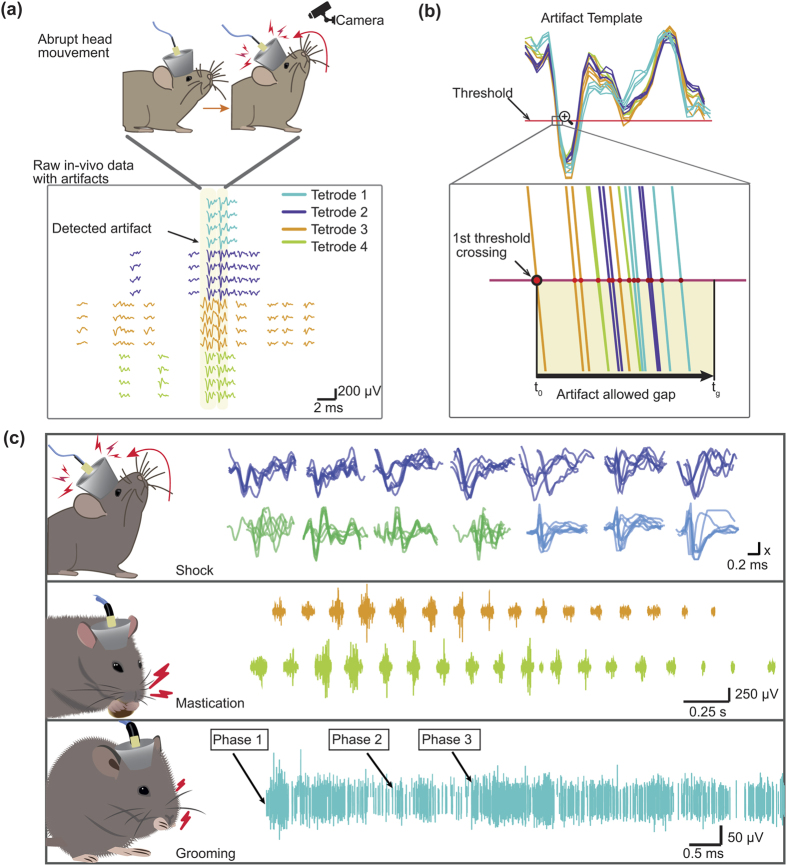
Artefact Library extracted from *in-vivo* real recordings. Artefact extraction is based on multiple validation criteria. (**a**) The artefact events were identified during unusual physical events captured by video monitoring (e.g. mechanical shock to the headstage, abrupt movement of the animal, grooming events, etc). (**b**) The artefacts were identified by their time-coincidence across channels (>80% of the total number of spatially distinct channels) within a width gap parameter (300 μs) starting from the first threshold crossing. (**c**) The library includes spike-like noise artefacts produced mainly by electronic interference or abrupt head movement, chewing artefacts and grooming artefacts. For the head collision artefacts each set shown in the figure is a superposition of waveforms corresponding to the first channel for each of the 7 tetrodes. The *x* value of the vertical axis of the scale bar is 20 μV, 30 μV and 40 μV for the blue, green and purple waveforms respectively. Mastication artefacts follow well identified rhythmic patterns (shown in Supplementary Fig. S2). Grooming artefacts appear across channels as high level movement artefacts with variable duration (See Supplementary Table 4).

For the artefact component of the benchmark generator, an artefact library was created by extracting artefact events from real data recordings^26^,^38^,^39^. The library contains spike-like sharp artefacts, grooming artefacts and mastication artefacts identified from different *in-vivo* extracellular recording experiments. The library was organised as indicated in Supplementary Fig. S1. The identification was made following multiple validation criteria stages that include: a test for simultaneous cross-channel appearance within an artefact gap of 300 μs in at least 80% of the total number of channels, a visual waveform inspection and a time-coincident comparison with simultaneous video recording (Fig. 4a and b), and a threshold crossing test for the spike-like sharp artefacts (thresholds selected are mentioned in Supplementary Table S1). Each artefact set *a*_*n*_ has between 22 and 32 template waveforms leading to identification and extraction of 40 mechanical shock artefact sets in 28 channels, 20 mastication artefact sequences in 32 channels, and 21 grooming artefact sequences in 28 channels.

The isolated mechanical shock artefacts are characterised by large peak-to-peak amplitudes (between 136.1064 μV +/−62.0262, see details in Supplementary Fig. S2 and Supplementary Table S2) and a peak frequency region around 1000–2000 Hz (Fig. 4c and Supplementary Fig. S2). These results are in line with a study that describes the characteristics of artefacts that are regularly found in *in-vivo* neural recordings^40^.

The mastication artefacts are electrical alterations of the recorded brain signals that appear during chewing events. Solid food provokes strong contractions of the jaw muscles which result in large rhythmic noisy bursts. This rhythmical oral behaviour is specific to mammals^41^ and can be identified across channels during electrophysiological recordings (See Supplementary Fig. S3). Characteristic rhythmic noisy bursts of the detected chewing events from rat recordings presented a mean chewing rate of 6.17 bursts/s with a mean duration of 3.3 s and a mean chewing cycle duration of 162.5 ms (see Supplementary Table S3). The identified mean chewing cycle duration results are consistent with previous studies in rat^41^.

Grooming artefact sequences across channels were extracted from recordings in mice in a task where they were allowed to groom freely^42^. Identified grooming artefacts appear across channels with large amplitudes and a heterogeneous duration range of ~0.4–28 s. The grooming events identified (phases 1 to 4) constitute a flexible grooming chain. The beginning of each phase of stereotyped movements was annotated within the artefact sequences (Fig. 4c and Supplementary Table S3).

**Figure 5:**
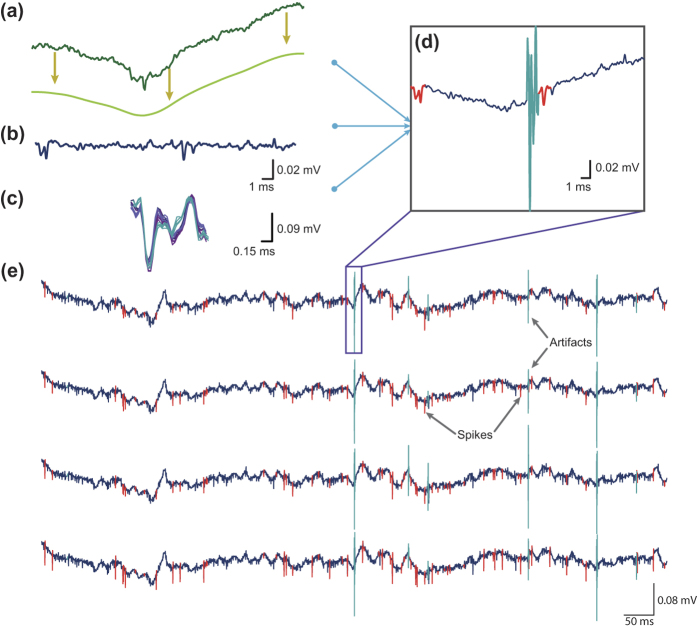
Generation of a ground-truth database of extracellular simulated realistic recordings. (**a**) Low frequencies extracted from real recordings using a Low Pass Butterworth Filter; (**b**) Neural data simulated from compartmental models and spike templates. (**c**) Annotated artefacts. (**d**) The addition of these three elements forms a completely parameterised benchmark. The action potentials are displayed in red and the artefacts in light blue.

The complete process of database generation is summarised in Fig. 5 and consists of the summing of the following three components: (1) non-stationary low frequencies, (2) annotated and parameterised action potential simulations and (3) the addition of identified artefacts.

### Generation of bio-realistic hippocampal benchmark databases in different experimental conditions

To challenge the accuracy and the performance of our model, we aimed to reproduce two types of real hippocampal extracellular multi-unit recording: in awake and in anaesthetised rodents. To mimic the macroscopic population activity of hippocampal neurons used in the real recordings, we set common parameters for both experimental conditions (i.e. those related to the recording environment such as the selected array of electrodes and those related to the simulation environment such as population density) but we differentiated two input parameters for the simulator that best approximated the dynamics of neural populations in our two distinct cases: firing rate and percentage of active neurons (Table 2 summarises the parameters chosen).

Our simulations reproduced neural signals acquired from the hippocampal layer CA1 region^14^,^26^,^34^,^35^. Different numbers of artefacts were assigned to the neural signals according to each experimental condition since recorded signals in anaesthetised animals tend to be less contaminated by artefacts than in freely behaving animals. For a 10 s simulation, an artefact rate of 1% of the signal was set for the awake condition and 0.1% for the anaesthetised case.

**Figure 6:**
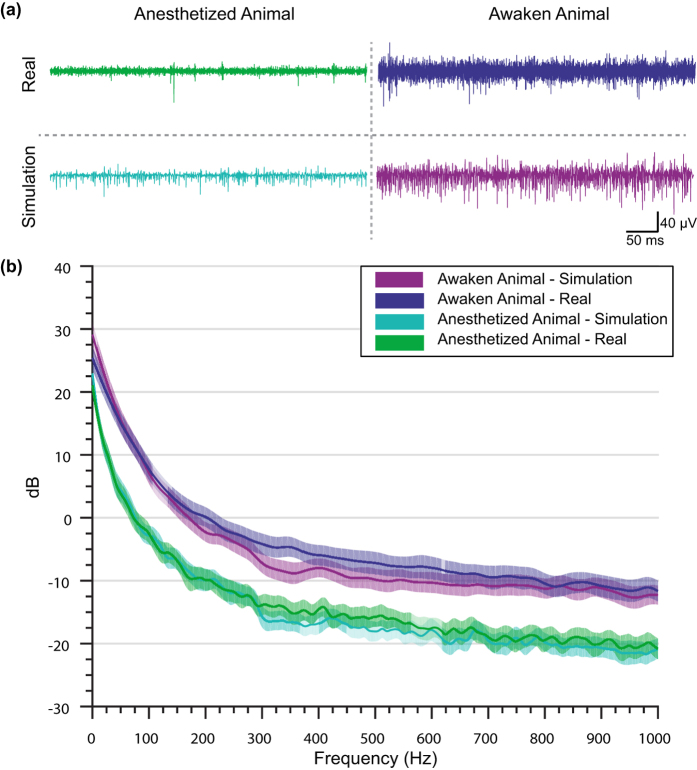
Inspection of simulated and real extracellular traces. (**a**) Qualitative comparison between real (600 Hz high-pass filtered) and simulated signals. (**b**) Averaged periodogram PSD estimate vs frequency for the real and simulated versions of the anaesthetised and awake versions of experiments. Low frequencies were extracted from different windows in the same recording session and linearly added to the simulated neural signals.

To assess the quality of our benchmark generator, we compared our simulated signals to real recordings. A time-domain examination showed that real and simulated signals had similar profiles in terms of amplitude and action potential distribution, for the two experimental conditions (Fig. 6a). We computed the averaged periodogram of the power spectrum density (PSD) estimate based on simulated and real signals of anaesthetised and awake rodents. The results confirmed that the distribution of power versus frequency components of the recorded signal in anaesthetised or awake animals were accurately reproduced by our model since no difference could be detected between real and simulated signals (Fig. 6b).

Interestingly, we could illustrate the utility of our parameterisable benchmark generator by looking at the effect at different contamination levels on electrophysiological signals. We explored the effects of application of different perturbation levels of artefacts using our annotated datasets for the two scenarios, in anaesthetised and awake subjects.

**Figure 7:**
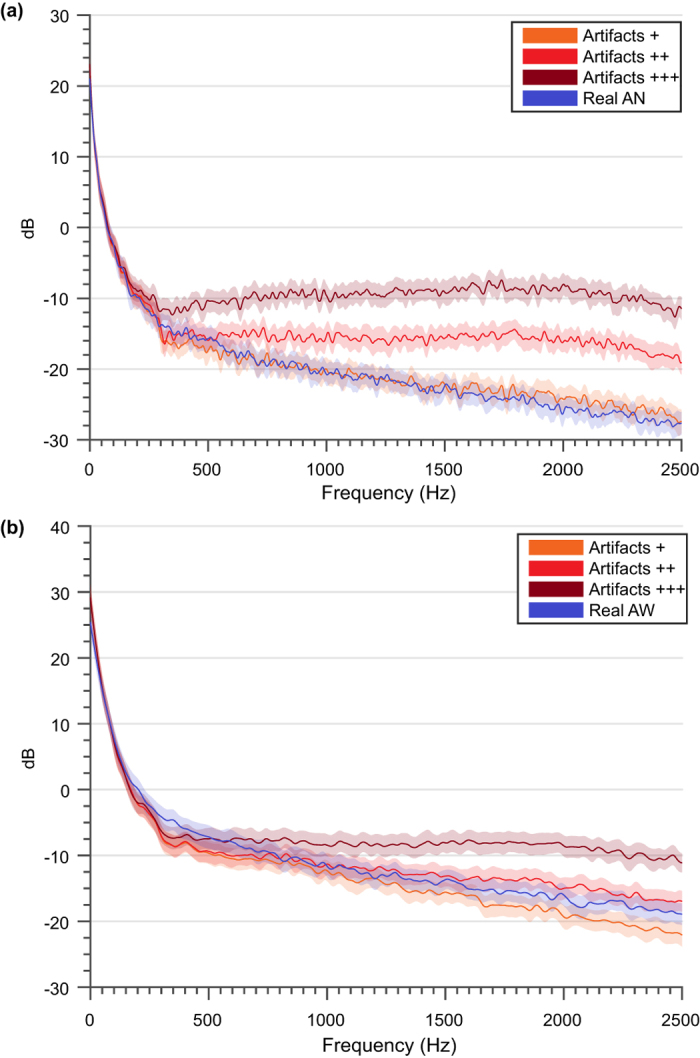
Averaged periodogram for simulations contaminated in different proportions. (**a**) Averaged periodogram PSD estimate vs frequency for the real and simulated versions of the anaesthetised paradigm. (**b**) Averaged periodogram PSD estimate vs frequency for the real and simulated versions of the awake paradigm. The signals (with a duration of 10 s) were contaminated with different artefact rates, starting at a contamination percentage of approximately 1% (+), subsequently 10% (++), and 57% (++) of the signal.

Our model predicted that the amount of artefact contamination would differentially affect the extracellular signals from anaesthetised or awake conditions. The evidence shows that, for the same level of signal contamination; the power-spectrum distribution was altered more in the anaesthetised than in the awake condition (Fig. 7). Action potentials contain a wide range of frequencies^36^,^43^ and the inherent higher frequencies overlap with the high frequency content of sharp artefacts which causes a growth in terms of power content in those frequency bands (as shown in Fig. 7). This phenomenon is more obvious for narrow extracellular spikes and it is easier to observe in contaminated recordings of anaesthetised animals, where the neural activity is lower than for the awake subject experiments.

The results confirmed that spikes and artefacts can be confused, both in amplitude and in frequency content. Thus, multiple testing that relies on other parameters should be taken into account to differentiate them, such as the extracellular spike width, wave shape and time appearance across channels.

## Discussion

We are currently witnessing an exponential increase of neural data collection paradigms with massive simultaneous recordings brought forward by the progress of microfabrication techniques and integrated sensors. The collection and use of such large amounts of neural information has stimulated the development of a number of hardware and software tools. Examples are signal acquisition devices, signal processing algorithms, or software for the calibration of brain-computer interfaces. To date, despite the necessity of benchmark datasets to test these kind of applications, there are surprisingly few ground-truth datasets available, and most of these are not parameterisable. Thus, there is an urgent need of such benchmarks to assess the validity of recently developed toolboxes and algorithms aiming to analyse neural data. Evidence of this need are initiatives such as the Spike Sorting Evaluation Project^44^, which aims to gather different benchmark datasets used to compare and evaluate software.

To address this issue, we developed a bio-inspired computational approach to create annotated and parameterised databases of neural signals. The innovative aspect was to combine neural signals simulated by a hybrid model with other components encountered in real recording such as artefact events and low frequency oscillations. To illustrate the flexibility of our methodology, we simulated two distinct experimental conditions; extracellular signals extracted from anaesthetised or awake rodents. We challenged our generated benchmark dataset by comparing the simulated signal with real experimental recordings.

Our results showed that the synthetic signals generated bore a close resemblance in terms of frequency properties and spike proportions to the recorded ones, and this held for our two different conditions. We showed that the addition to the simulated signal of common features encountered in real recordings (such as low frequency oscillations and artefacts) could have a significant impact on the spectral signature. Indeed, we found that the artefacts extracted tend to have a wide spectrum with dominant content at high frequencies that overlaps the neural spikes. These artefacts affect the frequency components in neural signals in different ways according to the percentage of the contamination of the signal and to the nature of the experimental setup (Figs 6 and 7).

Spectral analysis of our artefact library showed that most of the power of the signal from these events fell within the frequency range of 1000–3000 kHz. These values are similar to those shown for the power spectral density of action potential events^36^. Taken together, these results showed that the addition of artefact events into simulated signals, an innovation of our benchmark generator, is an essential component to consider as they can drastically corrupt the frequency domain signature of spiking activity.

Additionally, we showed an application example where we simulated a polytrode array across different virtual layers of tissue. Here the aim was to demonstrate how different experimental setups could be configured independently using the same simulator and how the different generated simulations accurately captured the overall neural activity.

One application where our benchmark generator could be of great interest is for testing devices and analysis modules used in closed-loop experiments, in which a stimulus is delivered immediately after a feature of interest is detected. In this configuration, a series of devices and software analysis modules interact to form the closed-loop chain. Between the key elements of the chain, online sorting algorithms and on-chip real-time modules (e.g. Field Programmable Gate Arrays (FPGAs) and Complex Programmable logic devices (CPLDs)) are key elements for online analysis. To correctly evaluate and compare the performance of these systems, the use of reliable benchmark datasets, such as the ones presented here, are essential. Ideally, this should be done by generating the datasets via the simulator and streaming them directly to the acquisition systems.

The datasets generated could be useful to evaluate the performance of various tools such as denoising and pattern recognition modules or spike sorting algorithms, implemented either in hardware or software.

In the future, this fully annotated benchmark should be optimised to fit more experimental scenarios. Some parameters and features could be added or replaced depending on the experimental conditions and the cellular and physiological properties of the neural substrate chosen for simulation. For example, in our model, irregular interspike intervals reflect a random process bounded between a predefined firing-rate distribution. However, in real recordings, it is common to find some neurons that fire action potentials in a bursty mode^45^. This feature could be added to the model by replacing the instantaneous firing rate with a generated probability distribution train of burst events.

One of the most challenging features to reproduce in synthetic signals is the background noise, given that there are many factors that shape it. Such disturbances can proceed from the subject itself (e.g. physiological background noise produced by the subject’s activity, additive and variable sources of current from other cells that are capture by the electrodes), the recording site (e.g. dimension, neural density, whether it be a preparation or not), the electronic instrumentation and the electrodes that couple to the tissue (e.g. thermal noise, shot noise, dielectric noise), external sources (e.g. electromagnetic and electrostatic coupling between the circuitry and external devices), and from the digital conversion itself (e.g. aliasing). Although there are metrics to measure their average contribution, it is still a major challenge to replicate every source of noise. We present here a library of common artefacts found during recordings that can be used to complete the benchmark datasets.

## Methods

In this section, we describe in detail the three main components of our benchmark generator.

### Hybrid model for neural signal simulation

For our experiments, we fixed the parameters that define the recording environment for both setups: the simulation model was a 1.5 *mm*^3^ cube with known randomly placed neurons with 16 recording sites and an electrode diameter of 13 μm. We considered a population density of 300,000 *neurons*/*mm*^3^ 46,47 for hippocampal neuronal density and a ratio of 80% pyramidal cells and 20% interneurons^48^.

We set firing rate ranges based on previous studies^49^,^50^ for the anaesthetised and the awake cases, respectively. Firing rates of interneurons were set by multiplying pyramidal cell average firing rates by a factor of five^51^,^52^, both for close and distant interneurons. The irregular interspike interval was defined by a uniform distribution bounded between a minimum and a maximum firing rate, respecting a refractory period. For both cases, anaesthetised or awake subjects, the refractory period was set at 2 ms, the sampling rate was 20 kHz and the total duration of each simulation was 10 s. The spatial distribution of the recording sites for the simulations presented here are illustrated in Supplementary Fig. S4.

Concerning our experiments, the level of artefact contamination of the signal was distinguished for the two experimental conditions, with 0.1% and 1% of the simulated signal contaminated in the anaesthetised and the awake animals, respectively. The number of artefacts for each channel recorded was defined by equation (2):


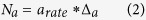


where *a*_*rate*_ is the average number of artefacts/s and Δ_*a*_ is the recording duration in seconds.

For each artefact event, a sample is added to the beginning and the end of the artefact waveform by curve fitting linear interpolation in order to smoothly add this waveform to the neural signal, this is done as follows:


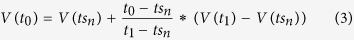


where *t*_0_ is the sample added to the beginning of the template, *t*_1_ is the sample where the template starts, *ts*_*n*_ is the sample immediately preceding *t*_0_ and *Vs* (*ts*_*n*_) corresponds to the value of that sample in *μ*V. The set of artefacts was integrated over time following a uniform random distribution that uses the Mersenne Twister algorithm^53^ to generate pseudo random numbers for a Uniform Distribution.

### Non-stationary slow oscillations

We used a 10^th^ order low-pass Butterworth filter applied in both the forward and reverse directions to maintain zero-phase distortion. In our design, the dataset used to extract the non-stationary slow oscillation component could be modified by the experimenter according to the nature of the signal intended to be simulated as well as the filter cut-off frequency. For our experiments, we used the real recording datasets previously reported^14^,^26^, low-pass filtered with a cut-off frequency of 300 Hz. Spike contamination in this frequency band was verified according to^37^ to have minimal effects on the extracted LFP. The resulting non-stationary extracted components were linearly added into the simulated signals.

### Artefact library

To extract the artefacts and create the library, we analysed neural data recorded from several different experiments. To detect artefacts, the signal had to cross the pre-defined amplitude threshold on at least 80% of the channels simultaneously (within an artefact window of 300 μs).

The head collision artefacts were recorded from mice during a behavioural task^42^. The original data consisted of seven single tetrode files recorded with a Cheetah160 Acquisition System with a total of 28 valid channels and a total recording duration of 3635.6 s. Each tetrode file is the result of a previous preprocessing analysis of the raw data, band pass filtered between 600–6000 Hz and a preset voltage threshold described in Supplementary Table S1. Individual waveforms were extracted and saved with their corresponding timestamps (Supplementary Table S1 shows the total number of detected waveforms for each tetrode file). Each waveform is 1142 μs in length with a pre-threshold period of 285 μs. The data was sampled at 28 kHz and stored at 32 points per waveform with their corresponding timestamp values and 16 bit A/D resolution.

The waveforms with shapes uncharacteristic of action potentials were marked as type 3 artefacts^40^ if they satisfied complementary verification methods (See Fig. 4). We created a library of 40 different sets of artefacts, where each set has between 14 and 18 artefacts recorded by the electrodes in ref. 42. In the simulation code we defined an average rate coefficient, that is, the number of artefacts/second of 1 and 10 for the anaesthetised and for the awake version of our simulations. The artefacts included present a distribution of amplitudes showed in Supplementary Fig. S2a.

To extract the grooming artefacts, the different grooming events were first identified from video recordings^42^. The different grooming phases were assigned according to a previous study^54^. Identified grooming sequences were paired to the simultaneous extracellular recordings for verification of the appearance of simultaneous artefacts across channels. The different grooming phases described in ref. 54 in the syntactic behavioural chain were annotated together with the artefacts (See Supplementary Table S4) in the library.

The chewing artefacts were extracted from electrophysiological recordings in rat. In this case, the animal was moving freely in a square arena chasing solid food rewards. We explored the recordings using NeuroScope software^55^ to visually identify abnormal augmented activity that stood out significantly from the background noise. We explored the data in the time-frequency domain and calculated the chewing cycle duration (1/mean chewing rate) and duration of the chewing sequence. We compared time-frequency analyses with the high-pass filtered data (300 Hz cut-off frequency) (See Supplementary Fig. S2).

### Real databases

The reference neural databases for real signals were recorded from separate groups of awake and anaesthetised animals^14^,^26^,^34^,^35^,^38^.

#### Case 1: Anaesthetised subjects

Real data consists of extracellular recordings in the hippocampus of anaesthetised rat^14^,^34^,^35^ with experimental procedures fully described previously^27^,^35^ and have been used by various laboratories as a benchmark for spike sorting algorithms. Animals (Sprague-Dawley rats) were anaesthetised with urethane (1.5 g/kg; Sigma). Extracellular electrodes were lowered into the CA1 layer of the hippocampus by monitoring for the presence of single unit activity.

#### Case 2: Awake subjects

The datasets include multichannel extracellular recordings from layer CA1 of the right dorsal hippocampus of Long-Evans rats during an open field task. In the task, the animal was placed on an elevated square platform and was looking actively for water rewards. Full details of the surgical and experimental procedures in awake recording were previously reported^26^,^45^ and are only briefly described here.

### Multi-layer simulation

The overall firing rate distributions of the SO, SP and SR layers were described in a previous study^45^ and reproduced here (see Supplementary Fig. S5), using the Matlab Distribution fitting tool^33^ with a logistic distribution and the following mean and scale parameters:


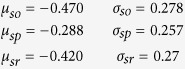


### Statistical analysis

We computed the Bartlett’s power spectrum density estimation (PSD) method^50^ of simulated and real signals to reduce the variance introduced by the periodogram while maintaining the frequency resolution^56^. The original benchmark datasets of 10 s duration were split into 10 non-overlapping 1 s length data segments. For each data segment we computed the periodogram using the discrete Fourier transform (see equation (4), where *s*_1,1_ is the data segment 1 from recorded signal from electrode 1).


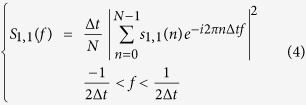


We then averaged the result of the periodograms for the 10 non-overlapping data segments:


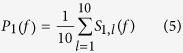


We finally computed the standard error of the mean by computing the segment standard deviation divided by the square root of the sample size. We performed the local fitting on the averaged result to smooth the data and used the weighted linear least squares and 2n degree polynomial method^57^ with a span of 1% of the data.

### Further information

Neural recording data from *in-vivo* rodents was used to assess the quality of our simulator. These datasets were available from previous studies which have been approved by the Institutional Animal Care and Use Committee of Rutgers University^34^,^35^. From these studies, we used the datasets “hc1”^14^ and “hc2”^26^ that have been made available to the community.

### Software access

Matlab code for generation and use of datasets described here, as well as the artefact library are available at http://bebgteam.net/resources. As previously described, the design is fully modifiable to simulate any specific experimental scenario that the experimenter wants to reproduce (e.g., amount of signal contaminated with artefacts, number of electrodes, distance between electrodes, etc.). To facilitate changes of the model, an XLM file and a Matlab configuration file are available where all the parameters can be rapidly modified.

## Additional Information

**How to cite this article**: Mondragón-González, S. L. and Burguière, E. Bio-inspired benchmark generator for extracellular multi-unit recordings. *Sci. Rep.* **7**, 43253; doi: 10.1038/srep43253 (2017).

**Publisher's note:** Springer Nature remains neutral with regard to jurisdictional claims in published maps and institutional affiliations.

## Supplementary Material

Supplementary Material

## Figures and Tables

**Figure 1:**
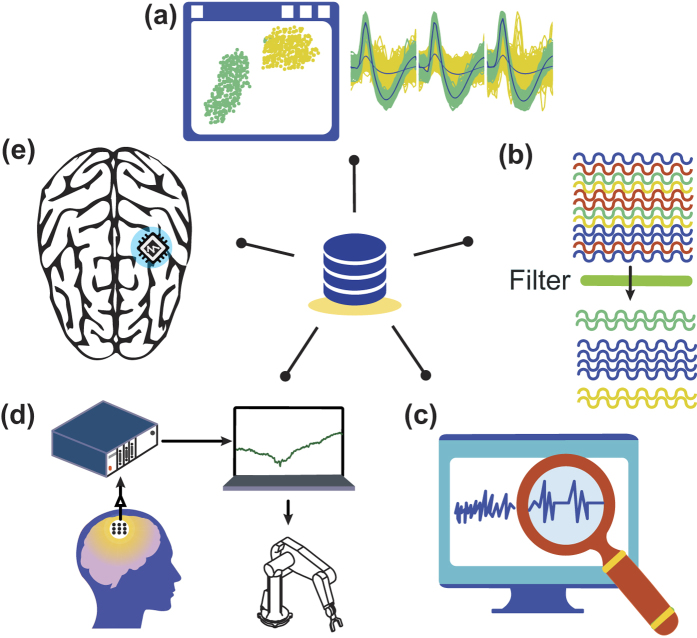
Examples of bio-inspired neural benchmark applications. Such benchmarks are needed in two contexts, on the one hand, in applications that involve fine signal processing usually executed on computers such as (**a**) neural pattern detection, (**b**) cluster classification algorithms and (**c**) signal denoising methods, and on the other hand in applications with direct exploitation of signals, usually executed on electronic devices, such as (**d**) brain-computer interfaces and (**e**) on-site decoding neural prosthetics.

**Table 1:** Simulation parameters selected for the multilayer virtual volume experiment.

Parameters	Stratum oriens	Stratum pyramidale	Stratum radiatum
Firing rate [*Hz*]^60^ *	~0.6 Hz	~0.4 Hz	~0.2 Hz
Percentage of active neurons^49^	10	10	10
Layer thickness [μm]	120	55	240
Population [neurons/mm^3^]^47^	11 300	272 400	1 900

A random distribution of point sources was set for this simulation. The refractory period was 2 ms, the sampling rate was 20 kHz. ^*^The firing rate for each layer followed a probability distribution defined in Supplementary Fig. S3 centred around a certain firing rate.

**Table 2:** Simulation parameters selected for both awake and anaesthetised experiments.

Parameters	Awake	Anaesthetised
Firing rate [*Hz*]^51^,^61^,^62^	0.5–12	0.5–5
Percentage of active neurons^49^	10	4
Population [neurons/mm^3^]^46^,^47^	300 000	300 000
% of artefact contamination	1	0.1

A random distribution of point sources was set for both simulations. The refractory period was 2 ms, sampling rate was 20 kHz and the simulation duration was 10 s.
