# Author Correction: Bio-inspired benchmark generator for extracellular multi-unit recordings

**DOI:** 10.1038/s41598-022-26829-0

**Published:** 2022-12-30

**Authors:** Sirenia Lizbeth Mondragón-González, Eric Burguière

**Affiliations:** Sorbonne Universités, UPMC Univ Paris 06, CNRS, INSERM, Institut du cerveau et de la moelle épinière (ICM), F-75013 Paris, France

Correction to: *Scientific Reports* https://doi.org/10.1038/srep43253, published online 24 February 2017

This Article contains an error in the Methods section, under the subheading “Software access”, where the hyperlink to external data is incorrectly given as http://bebgteam.net/resources. The correct hyperlink is https://gitlab.com/icm-institute/burguiere/publications/2017_scirep_mondragonburguiere.

