# Supplementary material for: Bio-inspired benchmark generator for extracellular multi-unit recordings
Source: Sci Rep. 2017 Feb 24;7:43253. doi: 10.1038/srep43253 (PMC5324125; doi:10.1038/srep43253)
Supplement: Supplementary Material [file srep43253-s1.pdf]

Supplementary Material

Bio-inspired benchmarks generator for extracellular multi-unit recordings

Sirenia Lizbeth Mondragon-Gonzalez[1], E. Burguière[1*]

[1] Sorbonne Universités, UPMC Univ Paris 06, CNRS, INSERM, Institut du cerveau et de la moelle épinière (ICM), F-75013 Paris, France

*Eric Burguière

eric.burguiere@gmail.com

1 Supplementary figures

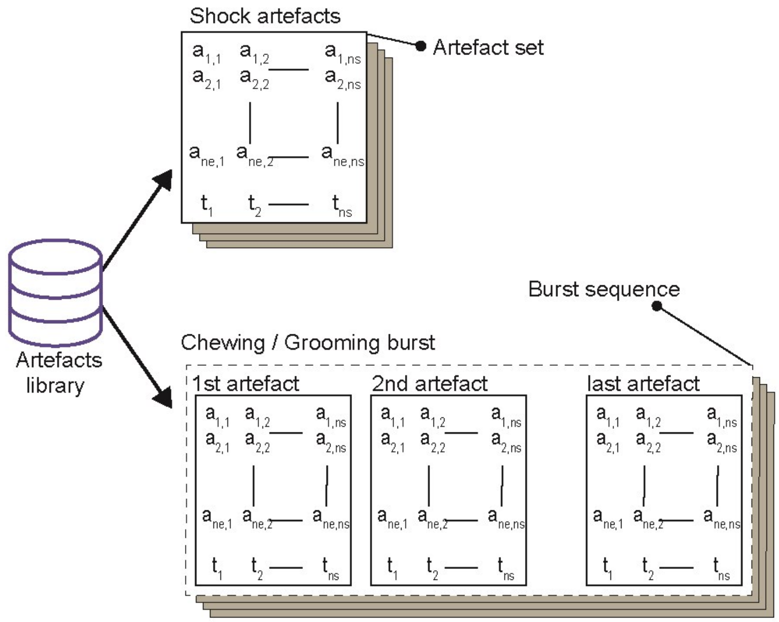

Supplementary Figure S1. Artefact library content. The artefact library contain spike-like artefacts or "head shock artefacts" that arise from headstage implant impacts to the electronics or from abrupt head movements, mastication artefacts and grooming artefacts. We stored the artefacts in artefact sets and artefact sequences that correspond to one single detected event. Every event has a different causal origin and this information is annotated for each set. A single artefact set contains an artefact event across channels. For the "head

impact artefacts" this is usually a sharp waveform and for the chewing and grooming artefact an artefact set contains one artefactual burst from a sequence. In the figure, $a_{x,y}$ is the value of the xth sample on the yth channel of the artefact, t_x is the timestamp of the xth sample. A single set contains minimum 22 artefacts and maximum 32 artefacts.

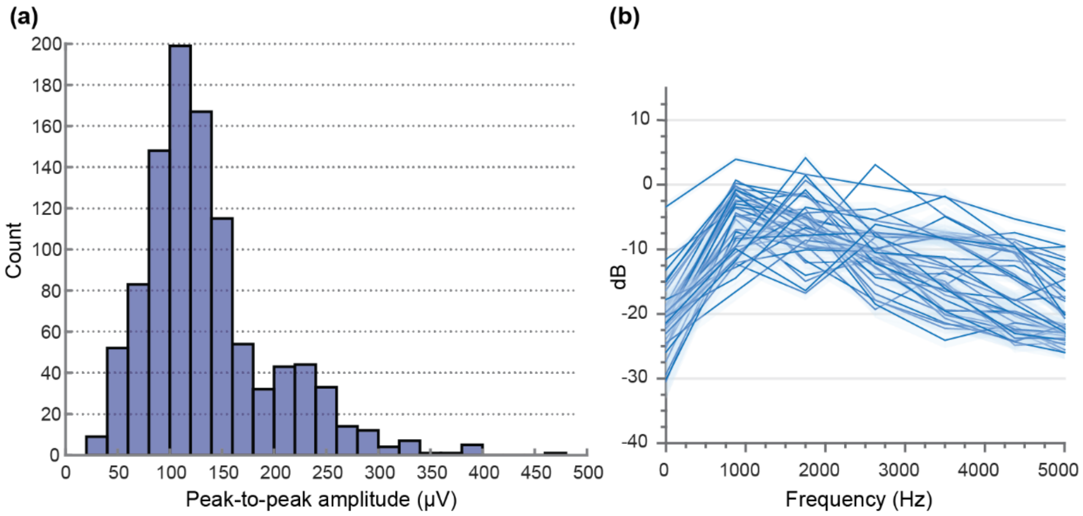

Supplementary Figure S2. Time-frequency analysis to identify signal artefacts of rodent chewing events. Characteristic rhythmic patterns of high-frequency and high-amplitude appear during mastication episodes. Trace (a) was recorded from one electrode during mastication, the chewing artefacts appear in red. In (b), the time-frequency analysis of (a) reveal the periodic bursts of chewing which are easily identified after high-pass filtering (in this case 300 Hz cut-off frequency) in (c). Bursts of chewing artefacts usually appear across channels as shown from the recordings in adjacent electrodes in panel (c).

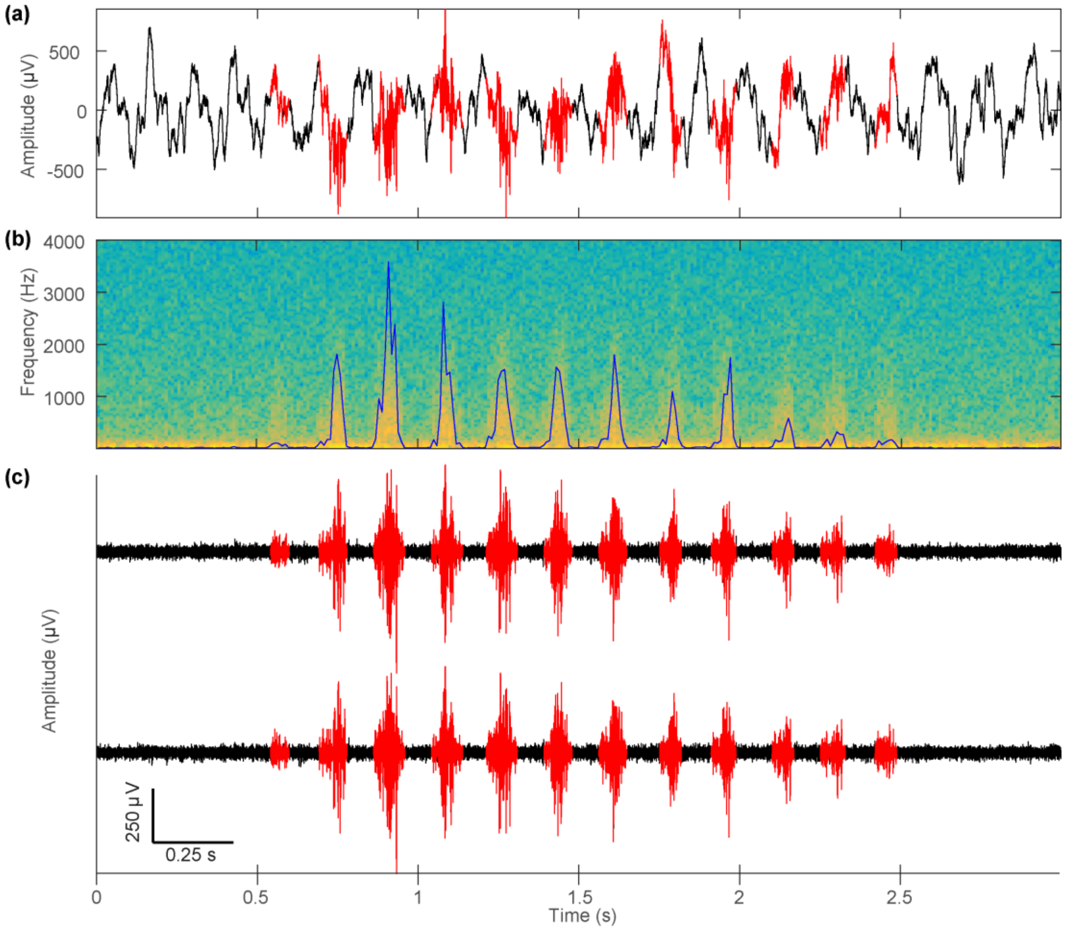

Supplementary Figure S3. Time-frequency analysis to identify signal artefacts of rodent chewing events. Characteristic rhythmic patterns of high-frequency and high-amplitude appear during mastication episodes. Trace **(a)** was recorded from one electrode during mastication, the chewing artefacts appear in red. In **(b)**, the time-frequency analysis of **a** reveal the periodic bursts of chewing which are easily identified after high-pass filtering (in this case 300 Hz cut-off frequency) in **(c)**. Bursts of chewing artefacts usually appear across channels as shown from the recordings in adjacent electrodes in figure (**c**).

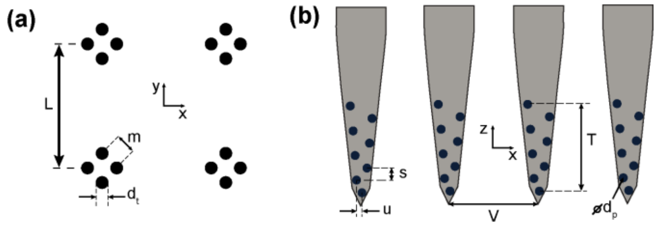

Supplementary Figure S4. Spatial distribution of virtual recording sites for the tetrode and polytrode configurations. All the parameters shown in the figure are easily modifiable within the coordinates configuration file. **(a)** The tetrode configuration array was defined by a distance between tetrode arrays “L”, and a inter separation distance “m”. For our application example values L=150μm, m=30μm and dt=20 μm **(b)** The polytrodes’ coordinates were defined by distance V=200μm, s=20 μm, T=120 μm and dp=15 μm.

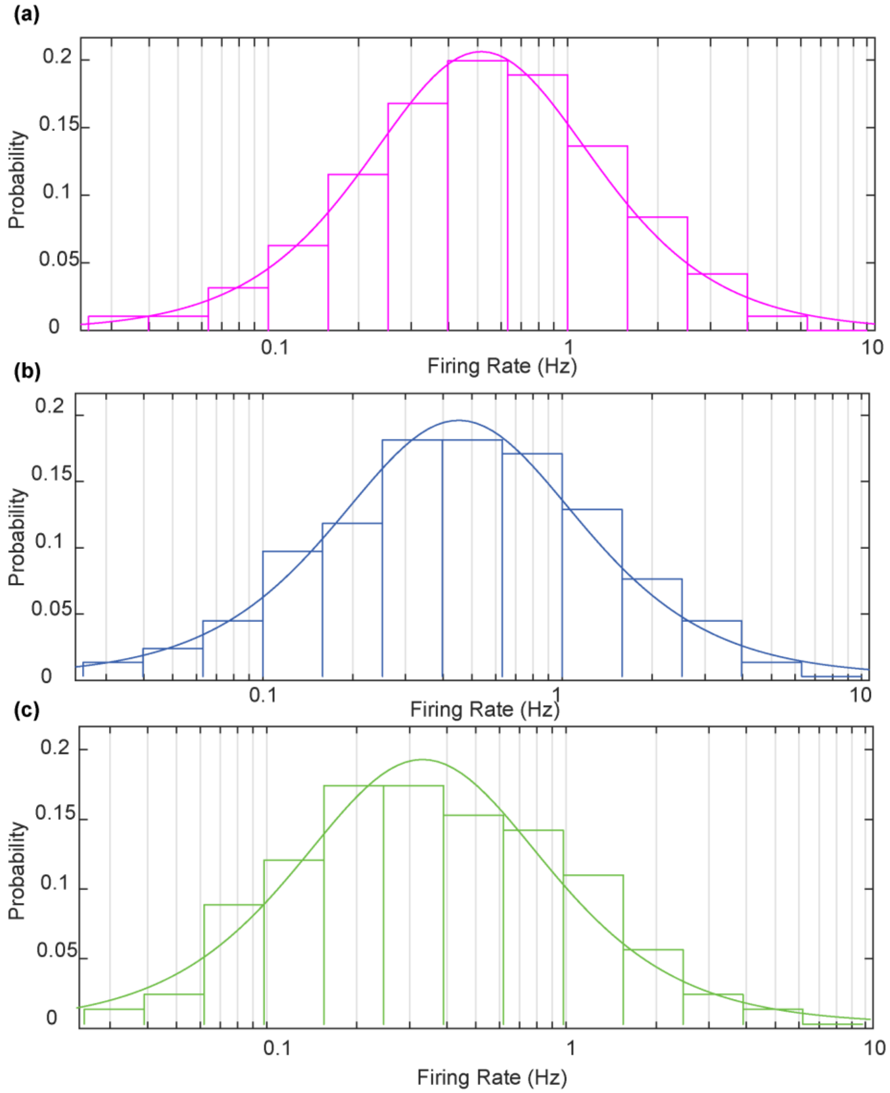

Supplementary Figure S5. Distribution of overall firing rates for each virtual layer. The distributions attributed to the different layers (**(a)** stratum oriens , **(b)** stratum pyramidalis, **(c)** stratum radiatum) follow the overall firing rates reported in a previous study[1].

2 Supplementary tables

Supplementary Table 1. **Waveforms summary.** It indicates the total number of waveforms for each tetrode. The total number of detected waveforms for all tetrodes is 1112953.

Tetrode number	Number of waveforms	Spike Threshold for channel 1 to channel 4 (µV)
1	68622	40, 40, 40, 40
2	363826	40, 40, 40, 50
3	125357	40, 40, 40, 40
4	44415	60, 60, 60, 60
5	127755	60, 60, 60, 60
6	326093	50, 50, 50, 50
7	56885	50, 50, 50, 44

Supplementary Table 2. Histogram bin counts. Peak-to-peak amplitude for the 1024 artefacts of the database. The bin edge is a uniform width of 20 µV.

Bin Edges (peak-to-peak amplitude in µV)	Number of waveforms	% of total number of artefacts
20-40	9	0.879
40-60	52	5.078
60-80	83	8.105
80-100	148	14.4530
100-120	199	19.434
120-140	167	16.309
140-160	115	11.23
160-180	54	5.273
180-200	32	3.125
200-220	43	4.199
220-240	44	4.297
240-260	33	3.223
260-280	14	1.367
280-300	12	1.172
300-320	4	0.391
320-340	7	0.684
340-360	1	0.098
360-380	1	0.098
380-400	5	0.488
460-480	1	0.098

Supplementary Table 3. Characteristics of chewing cycles detected. Duration, mean chewing rate across channels and chewing cycle duration (1/mean chewing rate) were calculated for each artefactual chewing sequence.

Sequence Number ID	Duration [s]	Mean chewing rate (CR) [bursts/s]	Chewing cycle duration [ms]
1	3.946	6.821	146.6061
2	3.8697	5.168	193.4985
3	3.3603	6.249	160.0256
4	3.9509	6.074	164.6362
5	1.5936	6.275	159.3625
6	2.9935	6.347	157.5548
7	2.746	6.554	152.5786
8	2.7998	6.071	164.7175
9	2.9121	6.524	153.2802
10	3.1005	6.128	163.1854
11	2.684	5.588	178.9549
12	2.426	6.183	161.7338
13	3.504	6.278	159.2864
14	4.169	5.756	173.7318
15	3.995	6.007	166.4724
16	3.388	6.198	161.3424
17	3.27	6.422	155.7147
18	3.6112	6.092	164.1497
19	4.474	6.348	157.5299
20	3.271	6.420	155.7632

Supplementary Table 4. Grooming artefacts.

**Sequence Number ID**	**Duration [s]**	**Phases identified in the syntactic chain**
1	7.9	1, 3
2	2	3
3	1.3	2, 3
4	0.4	1
5	2.2	1, 3
6	0.912	3
7	0.73	3
8	3.187	3
9	20.12	1, 2, 3
10	16.53	1, 3
11	10.96	1, 3
12	1.49	1, 3
13	0.612	1, 3
14	2.432	3
15	1.95	1, 3
16	17.91	1, 2, 3
17	7.68	1, 3
18	1.08	3
19	0.5	3
20	2.757	3
21	1.72	3
22	28.01	1, 3
23	3.536	3

Software

In order to create different simulations there are two files that can be rapidly modified:

- Coordinates.xml: to define recording sites' Cartesian coordinates and diameters.
- Configuration.m: to define the simulation parameters, dimension of the layers of virtual tissue and the population characteristics.

Main.m in the "Creation" folder will add the LFP and Artefacts from the library.
